# Mathematical modeling for glioblastoma treatment: scenario generation and validation for clinical patient counseling

**DOI:** 10.3389/fonc.2025.1647144

**Published:** 2025-09-29

**Authors:** Eric J. Kostelich, Yuan Xu, Carlos Calderón-Valero, Duane C. Harris, Oscar Alcantar-Garibay, Gerardo Gomez-Castro, Thomas J. On, Richard D. Dortch, Yang Kuang, Mark C. Preul

**Affiliations:** ^1^ School of Mathematical and Statistical Sciences, Arizona State University, Tempe, AZ, United States; ^2^ The Loyal & Edith Davis Neurosurgical Research Laboratory, Barrow Neurological Institute, St. Joseph’s Hospital and Medical Center, Phoenix, AZ, United States; ^3^ Department of Mathematics and Computer Science, Lawrence Technological University, Southfield, MI, United States; ^4^ Barrow Neuroimaging Innovation Center, Barrow Neurological Institute, St. Joseph’s Hospital and Medical Center, Phoenix, AZ, United States

**Keywords:** glioblastoma, mathematical modeling, personalized medicine, patient counseling, recurrent glioblastoma, tumor growth, neurosurgery

## Abstract

**Introduction:**

Glioblastoma (GBM) is an aggressive primary brain tumor. Despite standard treatment, recurrence is common, and patient counseling remains challenging. Mathematical modeling offers a potential strategy to simulate tumor behavior and personalize care. This study evaluates whether a simple reaction-diffusion model can generate realistic scenarios of treatment outcomes for individual patients with recurrent GBM using clinical imaging data.

**Methods:**

We retrospectively analyzed 132 MRI intervals from 46 patients who underwent treatment for recurrent GBM. T1 post-contrast and T2/FLAIR images were co-registered and manually segmented to identify enhancing tumor and edema. Using a systematic parameter sampling design, tumor growth between successive scans was simulated 18 times with a reaction-diffusion equation, the “ASU-Barrow” model, to generate realistic ranges of tumor response to treatment, as evaluated by clinical imaging.

**Results:**

Model-generated scenarios for changes in tumor volumes well approximated the observed ranges in the patient data. In 86% of the imaging intervals, at least one scenario yielded a simulated tumor volume that agreed to within 20% of the observed one (and to within 10% in 65% of the cases). Spatial accuracy was assessed using agreement and containment scores, indicating how well the predicted tumor matched the real one. The best simulations achieved an agreement of 0.52 and a containment score of 0.69. These results suggest that a simple model can generate a realistic range of outcomes, over intervals of two or three months, in a majority of patient cases.

**Conclusion:**

This reaction-diffusion model simulates likely ranges of GBM progression under treatment with reasonable accuracy and modest computational needs and may yield a clinically practical tool to support patient counseling. Incorporating advanced imaging, such as perfusion MRI, may further improve accuracy. With further development, our approach could provide personalized scenarios of treatment outcomes that could aid in patient counseling.

## 1 Introduction

Since the early twentieth century, mathematical models have been developed to understand tumor growth dynamics and optimize treatment strategies (1). The simplest population growth model is exponential: the population grows by equal ratios in equal intervals of time (e.g., the number of tumor cells doubles every week). The nineteenth century actuary Benjamin Gompertz proposed a modification in which the time intervals lengthen exponentially, so that the growth rate decelerates with time, reflecting resource limitations and, in the case of tumors, increasing cell death rates as the tumor becomes larger (2). The logistic model, also proposed in the 19th century, is a different modification of the basic exponential in which the growth ratio decreases linearly with the population size.

As oncology shifts towards individualized treatment approaches, some research efforts have explored the potential for mathematical modeling to optimize and personalize therapy. Examples include immunotherapy (3) and efforts to optimize regimens of chemotherapy and radiotherapy (4). Credibility assessments of computational models for medical devices and healthcare applications are an active area of research (5, 6). With respect to glioma specifically, Hormuth and collaborators have explored measures of tumor call heterogeneity (7) to predict future tumor growth and the use of imaging and mathematical modeling to predict treatment response (8). (Section 5 contains further discussion.)

Patients who are newly diagnosed with glioblastoma (GBM) typically are treated with maximal safe resection, radiotherapy, and temozolomide chemotherapy (9, 10). There is no standard of care for recurrent tumors (11, 12), but options include chemotherapy, radiation, electric fields, immunotherapy, and reoperation (13, 14). The choice of therapy depends upon tumor response and the patient’s performance status (15).

Nevertheless, there have been no treatment breakthroughs for GBM in the past 20 years, and improvements in patient survival in the interim are due largely to better supportive care (16). Patients with recurrent GBM face a poor prognosis, with tumor-related neurological decline and impaired quality of life as key concerns. Shared decision-making between patients, their families, and clinicians is important (17). Given the difficulties with the current state of the art, new tools are needed to assist with clinical counseling of patients.

We have undertaken this modeling study with a mindset like that of financial planners, who provide personalized estimates of portfolio performance to give clients an approximate idea of future retirement income. They do not attempt to model the global economy or predict the stock market. Instead, they use simple models (e.g., the time value of money) and sample prior rates of return in a statistically reasonable way to generate a range of plausible *scenarios*, based on a client’s current holdings, savings rate, and anticipated expenses. This procedure cannot account for every potential outcome, nor does it make specific *predictions*, but it can provide an idea of what to expect under certain hypotheses and serve as an advising tool.

In a similar vein, the objective of this study is to determine whether a simple mathematical model can simulate a personalized, realistic *range of potential outcomes* over the next 2 to 3 months, corresponding to typical follow-up intervals, for patients who experience a recurrence of their GBM tumors following initial treatment and whose response to future therapy cannot be predicted with certainty. To our knowledge, the potential for mathematical modeling to provide personalized scenarios of GBM progression in individual patients, which clinicians could use to assist with patient counseling, has not previously been assessed.

The biology of GBM is complex, and many details are poorly understood. Factors that may affect tumor progression and response to treatment include genomic abnormalities (18), neovascularization (19), hypoxia (20), immunosuppression (21), GBM stem cells (22), and the brain’s extracellular matrix (23), to name a few. A fundamental difficulty is that there is no established biochemical principle to justify a particular mathematical model of any of these processes and their interactions. Any associated rate equation is speculative at best and introduces coefficients and initial conditions that cannot be measured in living patients. Consequently, the biological correctness of the resulting model cannot be independently validated (24). A complex model is unlikely to be amenable to rigorous analysis, and its output may depend sensitively on parameters that cannot be estimated reliably (25), making it impractical for clinical application.

For these reasons, we focus on a model whose parameter space can be tractably sampled and that can be initialized from (and compared against) imaging data that are collected as part of routine clinical surveillance. We reiterate that we cannot make a specific *prediction* about a given patient’s clinical course; instead, our goal is to develop a data-driven modeling system, using a simple model and validated against the clinical trajectories of many previous patients, that can generate a personalized range of *realistic scenarios* of treatment outcomes. As noted above, our focus is on time horizons of 2 to 3 months, corresponding to typical surveillance imaging intervals. In this way, clinicians could use the outputs as a tool to help patients and their families make better-informed decisions about continued treatment.

## 2 Materials and methods

### 2.1 Patient population and data preprocessing

Magnetic resonance imaging (MRI) scans were obtained from the Barrow Neurological Institute (BNI) patient archive pursuant to St. Joseph’s Hospital and Medical Center Institutional Review Board (IRB) protocol PHX-19-500-182-20-08. They consist of 357 axial surveillance scans from 75 unique patients, ranging in age from 25 to 78 years (median 62), who previously had undergone maximal safe surgical resection, followed by radiotherapy and, in most cases, temozolomide chemotherapy. The first scan from each patient series is obtained 2 to 12 weeks after initial resection; each patient has at least 2 (and up to 9, median 4) scans that include T1 plus contrast and T2/FLAIR sequences. The median time interval between scans is 57 days (range: 16–121 days).

The imaging workflow proceeds as described in previous work by the authors (26) and consists of the following steps.

1. The patient imaging data consists of DICOM files generated by the MRI scanners. Identifying information is removed, and the data, consisting of axial slices, is assembled into a single three-dimensional image and converted to the NIfTI format (27) using SPM-12 (28); this is done independently for each T1 and T2 modality.
2. SPM-12 also is used to co-register all images to a common time point (usually the first scan in each patient series). The automated algorithms in SPM-12 are used to generate another NIfTI file that contains only the brain (the skull and eyes are stripped out), and the brain domain is further segmented into regions of cerebrospinal fluid (CSF) and white and gray matter. This labeled, patient-specific brain domain serves as the computational domain for each simulation. Altogether, it contains 24 to 30 axial slices of typically 256 × 256 voxels. The horizontal resolution varies from 0.85 to 0.93 mm and the vertical from 7 to 7.5 mm, depending on the number of slices in the original scans.
3. All MRI scans are manually segmented by neuroimaging experts. For this purpose, the co-registered scans are loaded into the 3D-Slicer image processing platform (29), which is used to “paint” the relevant voxels and separate them from anatomically normal tissue according to the neurosurgical judgment of the operator. Tumor voxels are segmented into three categories: necrotic core (hypointense on T1), enhancing tumor (contrast enhancement on T1), and tumor-associated edema (hyperintense on T2-FLAIR). The modeling software uses the segmentations to generate initial conditions, as described below.

For each patient, and at every time point, this procedure produces six or seven anonymized files in NIfTI format: the three-dimensional T1C and T2 images; the derived computational domain; and the manually produced segmentations of enhancing tumor, edema, resection cavity, and, if applicable, necrotic tumor core. All files are named and stored in a consistent way. A separate spreadsheet tracks the number of days between each scan, and a database, maintained at St. Joseph’s Hospital, links the numbered patients to the original scans and medical records.

Scans from the patient database are excluded from further analysis under any of the following circumstances:

- SPM-12 is unable to segment the brain domain (possibly as a result of motion artifacts in the scan), because we cannot construct a patient-specific computational domain;
- no enhancing tumor is apparent on T1 post-contrast imaging, because we cannot compute relative changes in tumor size; or
- the patient undergoes another surgical resection, which is beyond the scope of the model.

Following these exclusions, we have a complete set of preprocessed scans from 46 unique patients for 132 time intervals. The first two preprocessing steps outlined above can be done on a laptop in a few minutes, but the tumor segmentation is performed manually for reliability and thus is the most time-consuming step.

### 2.2 Mathematical model

Our modeling effort focuses on the gross total expansion (or contraction) of the tumor. Tumor volume is a prognostic factor for overall survival (30–32) and can be readily measured using the 3D-Slicer platform (33) from the surveillance imaging that serves as our data source.

The Fisher-Kolmogorov-Petrovsky-Piscunov (FKPP) equation is a reaction-diffusion equation that was proposed initially to model the spread of invasive species (34, 35). This model also has been proposed to describe the diffusive spread of GBM tumor cells throughout the brain parenchyma (36) and is given by


(1) $$\frac{\partial u}{\partial t}=\nabla \cdot \left(D\left(x\right)\nabla u\right)+\rho \left(x\right)u\left(1-\frac{u}{K}\right)$$

where u=u (x,t) is the space- and time-dependent tumor cell population, *K* is the local carrying capacity, and *D* and *ρ* are the diffusion and proliferation rates, respectively, which may be taken as constant or allowed to vary by location (e.g., faster diffusion in white matter than gray). Equation 1 admits traveling-wave solutions whose propagation speed depends on the model parameters, but the details also depend on the rates at which glioma cells switch between proliferative and motile phenotypes (37). Equation 1 has been suggested as a way to quantify the effect of surgical resection (38); the effect of chemotherapy (39); and to explain differences in patient survival after similar courses of treatment (40).

The model (1) assumes implicitly that all cells in the modeled population have the same growth potential. One characteristic of GBM tumors, however, is the presence of a hypoxic or necrotic “core” (41). The authors have previously proposed a modification of the FKPP equation (26) to account for this phenomenon, given by the so-called “ASU Barrow” (ASUB) model, which partitions the tumor cells into proliferating (*p*) and quiescent (*q*) subpopulations:


(2) $$\frac{\partial p}{\partial t}=\nabla \cdot \left(D\left(x\right)\nabla p\right)+g\left(p,q,t\right)-h\left(p,q,t\right)$$

(3) $$\frac{\partial q}{\partial t}=h\left(p,q,t\right).$$

The densities of proliferating and quiescent cells are given by the respective space- and time-dependent functions *p* = *p*(*x*, *t*), and *q* = *q*(*x*, *t*). The overall dynamics are simple: proliferating cells diffuse at a rate given by ∇ · (*D*(*x*)∇*p*); they grow an a net per capita rate *g*(*p*, *q*, *t*) and they become quiescent (i.e., die or become hypoxic) at a per capita rate *h*(*p*, *q*, *t*). The function *D*(*x*) defines the rate at which cells infiltrate the brain. Quiescent cells simply accumulate, reflecting high cellularity but no net tumor growth.

In Equations 2, 3, *h*(*p*, *q*, *t*) reflects the net cell-killing effect of treatment; *g*(*p*, *q*, *t*) captures treatment resistance and net proliferative tendencies. In the version simulated here, we take


(4) g(p,q,t)=ρ(x)p[1−δ(p+q)]

(5) h(p,q,t)=k(x)pδ(p+q),

where *ρ*(*x*) and *k*(*x*) are the space-dependent maximum growth and quiescence rates, and *δ* is a monotonically increasing function of the total cell density.

We take *δ*(*x*) to be piecewise constant: we fix one value in edematous tissue and a possibly different value in the enhancing rim of the tumor; similarly for *k*(*x*). One rationale is that some treatments, such as chemotherapy wafers or localized radiotherapy, may be applied (and have greater effect) on regions corresponding to contrast enhancement. We treat the diffusion rate analogously: *D*(*x*) = *D_w_* when *x* corresponds to a location in a white-matter tract or tumorous region; *D*(*x*) = *D_w_*/2 in gray matter; and *D*(*x*) = 0 in CSF.

We normalize the maximum cell density to 1 and require *δ*(0) = 0 and *δ*(1) = 1. In regions where the cell density is low, 1 − *δ* is close to 1; therefore, g≈ρp and h≈0 , so that the net proliferation rate is approximately exponential. In other words, small cell populations grow at a rate that is roughly proportional to $e^{\rho t}$ , which implies that the overall tumor growth rate can be sensitive to the choices of *ρ* in edematous regions, where tumor cell densities are presumed to be low. One convenient choice for *δ* is the beta cumulative distribution function *B*(*x*; *a*, *b*), which increases monotonically from 0 to 1 across the unit interval; we fix *a* = 3 and *b* = 1 (26).

### 2.3 Simulation of the model

The SPM-12 brain segmentation defines the patient-specific computational domain. No-flux boundary conditions are imposed at the interface with the skull and CSF. The model can be integrated efficiently using the IRKC solver by Shampine et al. (42). One 60-day simulation on a typical 256 × 256 × 25 patient brain domain takes about 18 seconds on a single CPU core of a modern personal computer. Multiple patient scenarios can be simulated in parallel on a multicore machine.

As described above, the growth and quiescence functions ρ(x) and k(x) in Equations 4, 5 are piecewise constant: $\rho \left(x\right)=\rho_{c}$ and $k\left(x\right)=k_{c}$ if *x* corresponds to a voxel in a region segmented as contrast enhancing, and $\rho \left(x\right)=\rho_{e}$ and $k\left(x\right)=k_{e}$ in edematous regions. To simplify their interpretation, the values for *ρ* and *k* are given in the tables below as doubling and halving times, respectively, in days, assuming pure exponential growth and decay, which occurs when the tumor cell populations are very small or large. (The actual parameters used in the model are $k=\left(\log2\right)/\hat{k}$ and $\rho =\left(\log2\right)/\hat{\rho }).$ The net doubling rate of glioblastoma tumor cells is not known, but one study estimates a tumor volume doubling time of 31 days prior to treatment (43) and another, a range from 14 to 49.6 days (44). A study of unresectable gliomas in children estimates tumor halving times of 60 to 78 days in response to radiotherapy and overall doubling times of 48 to 60 days for high-grade gliomas (45). Values of the diffusion rate *D*(*x*) also are unknown; one review of the literature has found published values that vary by four orders of magnitude [cf. Table 2 in (46)]. The FKPP Equation 1 predicts that tumors must reach a volume that is proportional to $\sqrt{D/\rho }$ to persist (47), and various studies have attempted to estimate *D*/*ρ* from MR imaging [e.g., (48)]. However, this approach is restricted to untreated tumors, as the prediction assumes a uniformly expanding cell population, and *D* and *ρ* are not identifiable from the ratio. Given the considerable uncertainties, the values of *D*, *ρ*, and *k* in **Table 1** are roughly consistent with the values in the cited studies.

**Table 1:** The 18 parameter sets used to simulate each tumor.

I	${\hat{k}}_{0}$	${\hat{\rho }}_{0}$	*D_w_*	*I_e_*	*I_c_*	${\hat{k}}_{1}$	${\hat{\rho }}_{1}$
1	14	21	0.015	[0.012, 0.03]	[0.16, 0.80]	18	27
2	35	35	0.030	[0.016, 0.04]	[0.22, 0.40]	37	27
3	56	49	0.060	[0.024, 0.06]	[0.30, 0.50]	58	27
4	56	49	0.030	[0.016, 0.04]	[0.16, 0.80]	18	57
5	14	21	0.060	[0.024, 0.06]	[0.22, 0.40]	37	57
6	35	35	0.015	[0.012, 0.03]	[0.30, 0.50]	58	57
7	56	35	0.060	[0.012, 0.03]	[0.22, 0.40]	18	97
8	14	49	0.015	[0.016, 0.04]	[0.30, 0.50]	58	97
9	35	21	0.030	[0.024, 0.06]	[0.16, 0.80]	58	97
10	14	35	0.030	[0.024, 0.06]	[0.30, 0.50]	18	27
11	35	49	0.060	[0.012, 0.03]	[0.16, 0.80]	37	27
12	56	21	0.015	[0.016, 0.04]	[0.22, 0.40]	58	27
13	35	49	0.015	[0.024, 0.06]	[0.22, 0.40]	18	57
14	56	21	0.030	[0.012, 0.03]	[0.30, 0.50]	37	57
15	14	35	0.060	[0.016, 0.04]	[0.16, 0.80]	58	57
16	35	21	0.060	[0.016, 0.04]	[0.30, 0.50]	18	97
17	56	35	0.015	[0.024, 0.06]	[0.16, 0.80]	37	97
18	14	49	0.030	[0.012, 0.03]	[0.22, 0.40]	58	97

The parameters are listed from left to right in deceasing order of sensitivity.

Initial conditions are imputed from the respective tumor segmentations. Chang and co-workers (49) found an approximate linear relationship between MR signal intensity and GBM cell density, with cellularity increasing with intensity in T1-weighted post-contrast imaging and decreasing with T2/FLAIR signal intensity. In our simulations, we ascribe an initial population within the interval $I_{c}=\left[p_{1}^{c},p_{2}^{c}\right]$ that increases linearly with T1C image intensity in regions segmented as enhancing tumor and decreases linearly within the interval $I_{e}=\left[p_{1}^{e},p_{2}^{e}\right]$ with T2/FLAIR signal in the edema segmentations. Counting the intervals *I_c_* and *I_e_* as one parameter apiece, there are 7 adjustable parameters, which are listed in decreasing order of sensitivity on the simulation results in **Table 2**.

**Table 2:** Model and initialization parameters for the tumor simulations.

Order	Parameter	Units	Description
1	${\hat{k}}_{0}$	d	max quiescence rate, edema
2	${\hat{\rho }}_{0}$	d	max growth rate, edema
3	*D_w_*	mm^2^ d^−1^	diffusion rate, white matter and tumor
4	*I_e_*	interval	population range, edema
5	*I_c_*	interval	population range, enhancing region
6	${\hat{k}}_{1}$	d	max quiescence rate, enhancing region
7	${\hat{\rho }}_{1}$	d	max growth rate, enhancing region

### 2.4 Experimental design

Of course, we do not know the “true” values for the model parameters and initial conditions, which probably vary considerably among the patients. Instead, our goal is to determine a likely range of values that yield clinically representative results. To make the computations tractable in such a large parameter space, we adopt a Taguchi experimental design, which is a type of Latin hypercube sampling. For simulation purposes, each parameter in **Table 2** is assigned one of three “levels,” corresponding to a “low,” “medium,” and “high” value. There are 3^7^ = 2187 possible combinations, but we choose only 18 by prioritizing the most sensitive parameters, ${\hat{k}}_{0}$ and ${\hat{\rho }}_{0}$ , and sampling according to an orthogonal array. The same set of 18 parameters is used to simulate every tumor. There are no stochastic components in the modeling framework presented here.

The orthogonal array consists of columns 2–8 in **Table 3** of Kacker et al. (50), which contains 18 rows. Simulations are run with each of the 9 possible pairs of values of the two most sensitive parameters, ${\hat{k}}_{0}$ and ${\hat{\rho }}_{0}$ . For each pair, two different choices of the remaining parameters are made by uniform sampling. The goal is to estimate the variability in the simulations as a function of the parameters in an economical way. **Table 1** displays each of the 18 parameter sets that are used to simulate each tumor.

**Table 3:** Observed and simulated chances that the patient’s tumor grows or shrinks (positive or negative percentages, respectively) by selected thresholds until the next scan time.

Change	Observed	Simulated
≤ −50%	10%	9%
≤ −25%	22%	19%
≤ −20%	25%	23%
≤ −15%	30%	27%
≤ −10%	37%	32%
*<* 0%	43%	45%
≥ 0%	57%	55%
≥ 10%	43%	41%
≥ 15%	38%	37%
≥ 20%	35%	33%
≥ 25%	33%	31%
≥ 50%	22%	21%
≥ 100%	14%	12%

The simulation code is highly parallelizable, and results for all of the parameter combinations can be obtained in about 80 seconds of wall-clock time on a Macbook Pro laptop for a typical 60-day imaging interval. We have also done 144 simulations according to a sampling design that uses 12 different levels of the parameters and obtained comparable results.

## 3 Results

Altogether, we evaluated tumor progression across 132 time intervals from 46 unique individuals with recurrent GBM. For each interval, we compare the number of enhancing voxels, *V*_0_, from the initial scan to the number *V*_1_ in the next, and define the relative change as


(6) $${\text{Δ}}^{\text{obs}}=\frac{V_{1}-V_{0}}{V_{0}}.$$

Notice that Δ^obs^ = −1 if there are no enhancing voxels in the comparison scan; Δ^obs^ = +1 if the number doubles; and Δ^obs^ = 0 if the number does not change.

**Figure 1:**
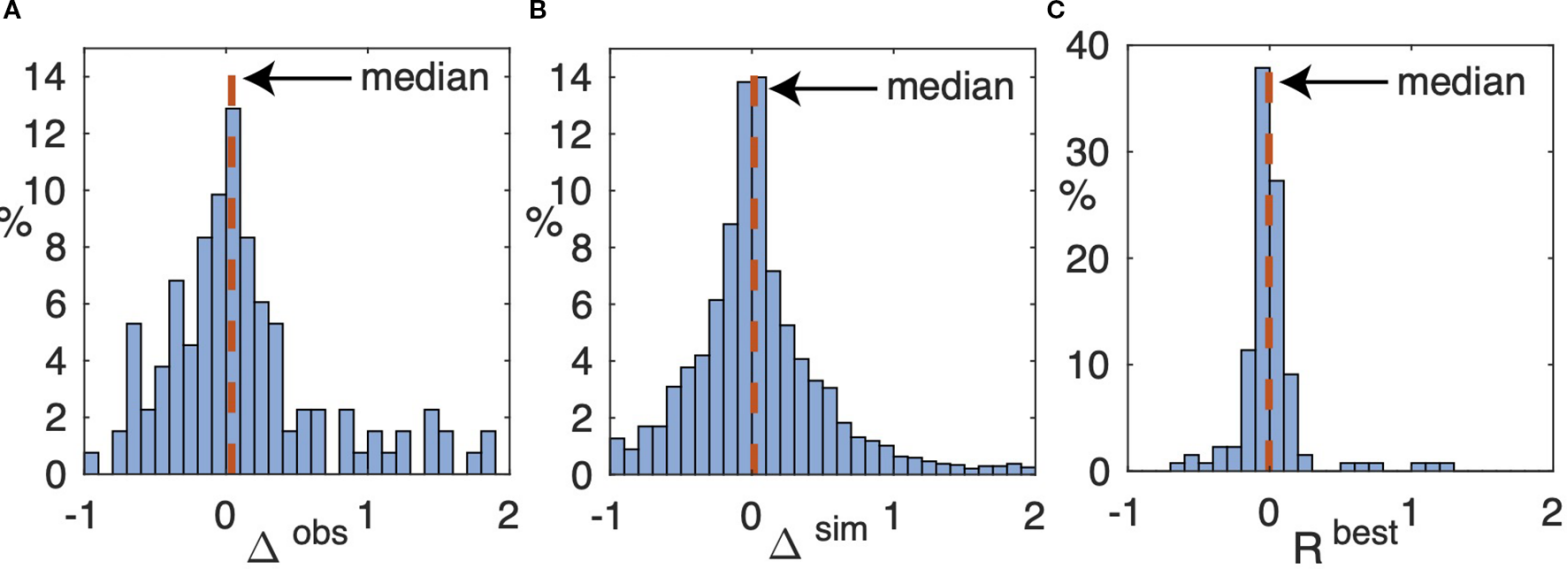
**(A)** Histogram of relative change Δ^obs^, Equation 6, of voxels in regions segmented as enhancing tumor between the starting and comparison scans over all 132 time intervals. Not shown are 6 cases (5%) where 2.22< Δ^obs^< 4.61 and the one case where Δ^obs^ = 21.29. The median, shown as a vertical dashed line, is 0.0377. **(B)** Histogram of the relative change, Δ^sim^, in all 18 × 132 simulated tumors over the same time interval. Not shown is the upper tail, containing 211 (8.6%) of the simulations, where Δ^sim^ *>*2 (the largest value is 74). The median is 0.0153. **(C)** Histogram of the relative change, *R* ^best^, defined in Equation 8 in the simulated tumors that most closely match the number of enhancing voxels in the corresponding comparison scans. Not shown is one simulation in which *R* ^best^ = 3.15. The median is −0.0049.

**Figure 1A** shows a histogram of the distribution of Δ^obs^ in the clinically observed tumors, which ranges from −0.917 (i.e., reduction of 91.7%) to an expansion by a factor of 21.28; the median change is 0.0377. (The horizontal axis is truncated at Δ^obs^ = 2 for ease of visualization.) There are 6 cases where 2< Δ^obs^< 5 and one case where Δ^obs^ *>*21.) In 75% of the cases, the observed changes range from 42.2% shrinkage to an expansion by about 111%.

The same set of 18 parameters, as listed in **Table 1**, is used to simulate each patient and imaging interval. In each simulation, we count the number of voxels in which the final tumor cell population, *p* + *q*, lies within the interval *I_c_*. For example, in the first parameter set, *I_c_* = [0.16, 0.80], so the voxels in which 0.16 ≤ *p* + *q* ≤ 0.80 are counted as enhancing. Likewise, if *p* + *q* lies within the interval *I_e_* = [0.012, 0.03], then the corresponding voxel is counted as edematous. (The calculation is similar for the remaining parameters.) Let $V_{i}^{\text{s}\text{i}\text{m}}$ denote this number for the *i*th simulation and define, for i=1,…,18 , the relative change in tumor volume as


(7) $${\text{Δ}}_{i}^{\text{sim}}=\frac{V_{i}^{\text{s}\text{i}\text{m}}-V_{0}}{V_{0}},$$

where *V*_0_ is the observed number of enhancing voxels at the start of the simulation interval.

**Figure 1B** shows a histogram of the distribution of Δ^sim^ for all 18 × 132 = 2376 simulated tumors. The distribution of Δ^sim^ is roughly consistent with the distribution Δ^obs^ of observed tumor volume changes, **Table 3** shows the likelihood that Δ^obs^ and Δ^sim^ falls within selected percentage ranges (negative percentages indicate the corresponding reduction in the number of enhancing voxels at the next scan time).

Each simulation produces 18 realizations (scenarios) of the evolution of the tumor. Of these, let *V* ^*^ be the volume of the realization that is closest to the volume *V* ^obs^ of the observed tumor at the next scan time. We define


(8) $$R^{\text{best}}=\frac{V^{*}-V^{\text{o}\text{b}\text{s}}}{V^{\text{o}\text{b}\text{s}}}$$

where *V* ^obs^ is the number of voxels in the subsequent scan that have been segmented as enhancing tumor.

**Figure 1C** shows the distribution of $R^{\text{best}}$ across all 132 simulated time intervals. In 86 (65%) of the cases, the relative error is less than 10%, and in 113 (86%), less than 20%. Furthermore, each of the 18 parameter choices produces a “best” simulated tumor for some patient imaging interval; none of the combinations in **Table 1** is redundant. These results, plus the threshold data in **Table 3**, suggest that the parameter ranges used in these simulations capture most of the likely variability in potential outcomes for typical patients. The relative errors exceed 100% in 4 (3%) of the cases (the largest is 315%, discussed in more detail below).

**Figure 2:**
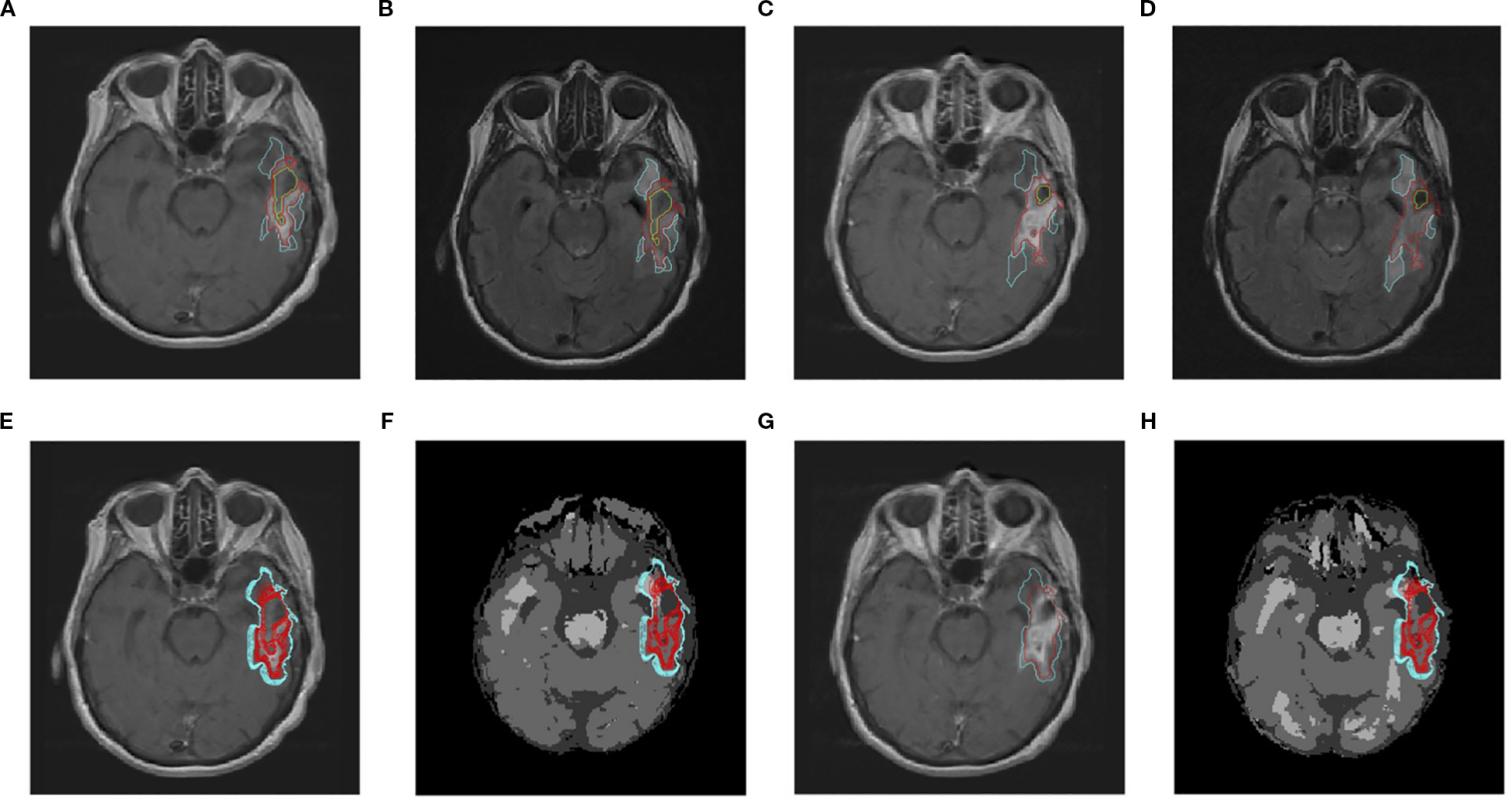
Representative simulation for a typical patient over an interval of 62 days, during which the volume of enhancing tumor grows from 1629 to 2175 voxels (33.5% increase). The axial slices shown encompass the approximate center of mass of the tumor. Top row: Patient scans and manual segmentations. Bottom row: Simulated tumor and computationally derived segmentations. **(A)** Initial post-contrast T1-weighted MRI. Cyan curves: edema segmentation; red curves: enhancing tumor. **(B)** As in **(A)**, but for the initial T2-weighted MRI. **(C)** As in **(A)**, but for the subsequent scan. **(D)** As in **(B)**, but for the subsequent scan. **(E)** As in **(A)**, but with the simulation ensemble superimposed. The red curves show the extent of the enhancing region for each of the 18 simulated tumors. **(F)** As in **(E)**, but superimposed on the computational domain produced by SPM-12. Dark gray: CSF; medium gray: gray matter; light gray: white matter. **(G)** As in **(C)**. Red curve: segmentation of the simulated tumor that most closely matches the observed number of enhancing voxels. Cyan curve: the corresponding simulated edema segmentation. **(H)** As in **(F)**, but superimposed on the brain segmentations produced by SPM-12 from the subsequent scan.

**Figure 3:**
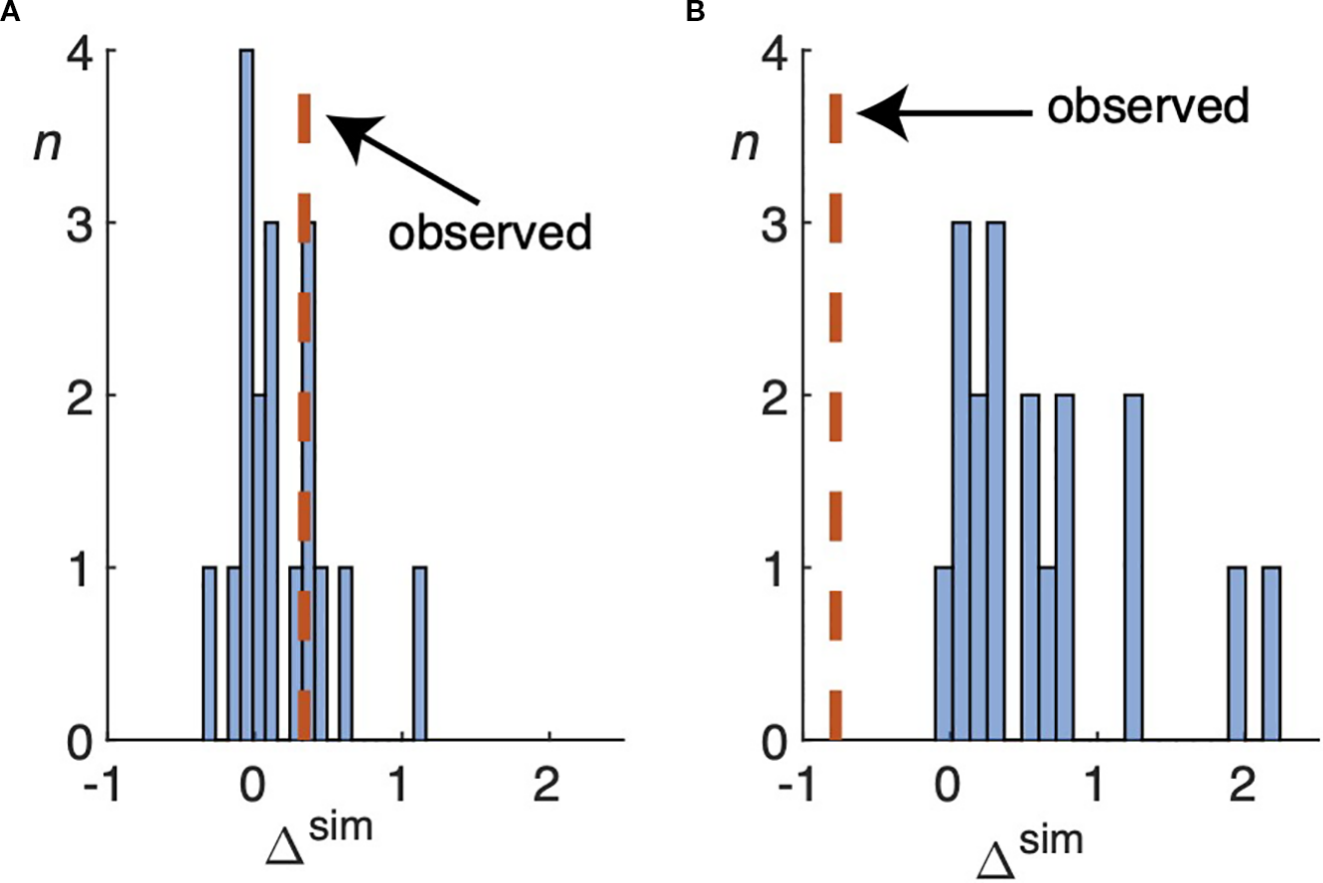
**(A)** Histogram of the simulated change Δ^sim^, defined by **Equation 7**, over all 18 parameter sets used to simulate the tumor for the patient in **Figure 2**. The vertical dashed line shows the observed change, Δ^obs^, as defined by Equation 6. In this case, the volume of enhancing tumor increases by approximately 33%. **(B)** As in **(A)**, except for the patient simulated in **Figure 5**. All of the parameter sets significantly overestimate the number of enhancing voxels in the comparison scan, which shrinks by about 78%.

**Figure 2** shows a representative simulation result. In this example, the actual tumor grows from 1629 to 2175 enhancing voxels, i.e., the observed growth, as defined in Equation 6, is Δ^obs^ = 33.5%. The ensembles produce simulated tumors that range from 1169 to 3495 voxels; the “best” parameter set produces a tumor with 2167 voxels, so the relative error, $\left|R^{\text{best}}\right|$ , is approximately 0.37%. **Figure 3A** shows a histogram of the simulated changes Δ^sim^, Equation 7, of the patient’s tumor for each of the 18 parameter sets. The observed change in tumor volume, Δ^obs^, is well approximated by several sets of the parameters.

Because the location of the tumor also matters, we define two spatial error measures, as follows. Let *R* denote the set of voxels in the region segmented as enhancing tumor in the actual patient scan and let *S* be those of the simulated tumor. We define |S| and |R| as the number of voxels in the respective regions. Then $\left|S\cup^{R}\right|$ is the number of voxels occupied by one or the other and $\left|S\cap^{R}\right|$ is the number of voxels in the overlap. We define the *containment* as


(9) $$C=\frac{\left|S\cap^{R}\right|}{\left|R\right|}.$$

and the *agreement* as


(10) $$A=\frac{\left|S\cap^{R}\right|}{\left|S\cup^{R}\right|}.$$

**Figure 4:**
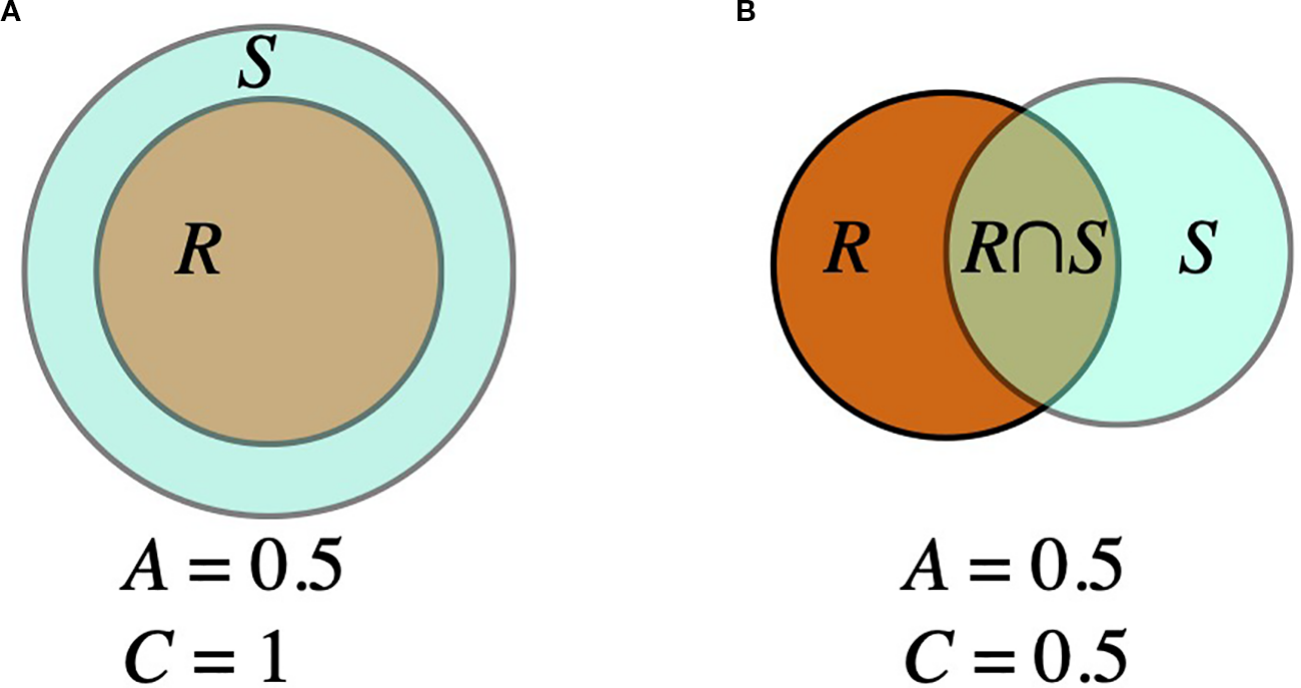
Two examples of the agreement (*A*) defined i Equation 10 and containment (*C*) defined in Equation 9 measures. **(A)** If *S* is twice as large as *R* but completely overlaps *R*, then *C* = 1 but *A* = 0.5. **(B)** If *S* and *R* are the same size but their overlap is only half of the area of each, then *A* = *C* = 0.5.

**Figure 4** shows an example of the two measures. A perfect model would produce *A* = *C* = 1 if the brain domains were exactly co-registered.

We compute the agreement measures by superimposing the simulated tumor on the computational domain derived from the subsequent scan. We compare the sets of voxels segmented as enhancing tumor in the subsequent scan with those from the simulated tumors (there are 18 such comparisons, one for each parameter set). The agreement measures range from 0.205 to 0.514 and the containment measures, from 0.408 to 0.799. The parameter set that most closely approximates the actual enhancing tumor volume gives agreement and containment measures of 0.466 and 0.634, respectively, in **Figure 2**.

Co-registration errors between successive patient scans are inevitable. To help interpret the agreement and containment measures from the simulations, it is useful to compare the corresponding measures between the brain segmentations derived from SPM-12 (cf. panels (f) and (h) in **Figure 2**). In this case, the respective agreement measures between the regions segmented as gray and white matter are 0.570 and 0.587 and the containment, 0.714 and 0.835.

**Figure 5:**
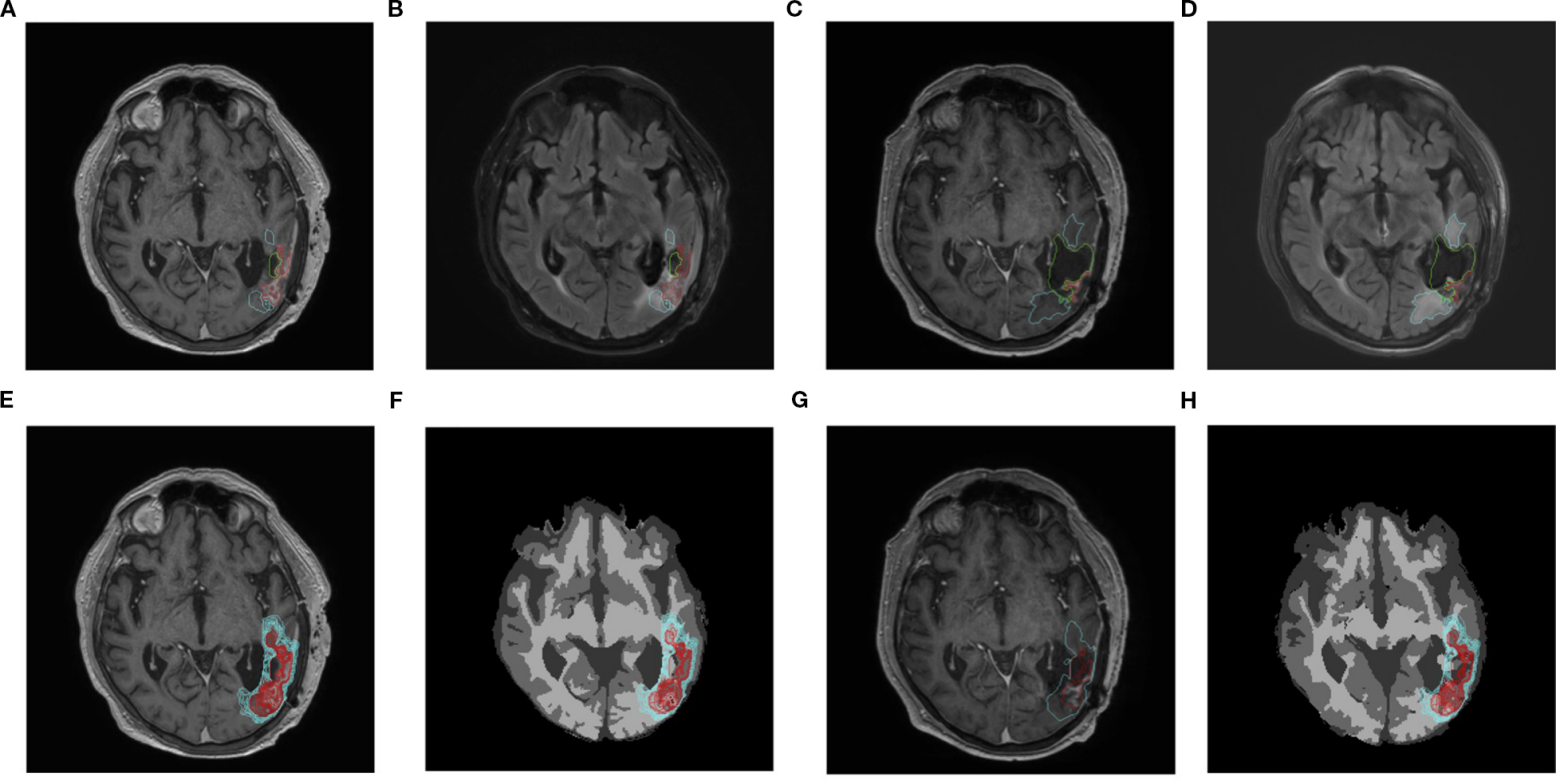
Results of the simulation with the largest relative error in enhancing tumor volume. The enhancing volume in the actual patient scans has decreased from 2245 voxels in panel **(A)** to 500 voxels in panel **(C)**. over an interval of 75 days. The axiel slices shown encompass the approximate center of mass of the tumor. Top row: Patient scans and manual segmentations. Bottom row: Simulated tumor and computationally derived segmentations. **(A)** Initial post-contrast T1-weighted MRI. **(B)** As in **(A)**, but for the initial T2-weighted MRI. **(C)** As in **(A)**, but for the subsequent scan. **(D)** As in **(B)**, but for the subsequent scan. **(E)** As in **(A)**, but with the simulation ensemble superimposed. **(F)** As in **(E)**, but superimposed on the computational domain produced by SPM-12. Dark gray: CSF; medium gray: gray matter; light gray: white matter. **(G)** As in **(C)**. Red curve: segmentation of the simulated tumor that most closely matches the observed number of enhancing voxels. Cyan curve: the corresponding simulated edema segmentation. **(H)** As in F(F0, but superimposed on the brain segmentations produced by SPM-12 from the subsequent scan.

**Figure 5**, which is organized identically to **Figure 2**, shows the results of the simulation with the largest relative error among the 132 that were performed for this study. In this case, the clinically observed volume of enhancing tumor has shrunk considerably, from 2245 to 500 voxels (Δ^obs^ = −0.773). The 18 simulated tumors (second row) range from 2075 to 7246 enhancing voxels. **Figure 3B** shows a histogram of all the simulated changes Δ^sim^, none of which well approximates the observed one; $\left|R^{\text{best}}\right|$ , is approximately 312%. The tumor agreement and containment measures range from approximately 0.02 to 0.18.

## 4 Discussion

This preliminary study shows the potential utility of simple mathematical models for generating scenarios of treatment response on an individualized patient basis using imaging data that is collected as part of routine clinical surveillance. The experimental design provides reasonably accurate estimates of the likelihood that a patient’s tumor grows or shrinks by a given threshold. At least one choice of parameters from the 18 used to simulate each tumor generates a result whose volume lies within 20% of the observed tumor in 86% of the time intervals studied. As illustrated in **Table 3**, the simulations match, within a few percentage points, the observed chances that a given patient’s tumor grows or shrinks by specified thresholds by the next scan time. The simulations and visualizations can be run on a laptop computer within a couple of minutes in a clinical setting. This section briefly addresses some related work as well as limitations and potential improvements to the present study.

### 4.1 Relation to prior work

Many mathematical models have been proposed to approximate the multiscale aspects of the growth of infiltrating gliomas, including glioblastoma (GBM) tumors. Most of them use reaction-diffusion equations (51), similar to the models presented here. Konukoglu et al. (52) proposed a method to estimate “patient-specific” parameters using a sequence of brain images. Hogea et al. (53) proposed a different scheme (with similar objectives) that also attempts to incorporate the tumor mass effect. While not yet standard clinical tools, some models have incorporated angiogenesis and oxygen transport to improve predictions of tumor dynamics (54, 55). Mostaghi-Kashanian et al. (56) proposed a reaction-diffusion model to distinguish visible and invisible tumor regions. Lipková et al. (57) presented a patient-specific model that links glioma growth with pressure distribution in the brain to estimate intracranial hypertension, midline shift, and cognitive impairment.

Several research groups have proposed mathematical modeling frameworks to optimize treatment strategies for GBM. One notable example is the study by Dean et al. (58), which developed a novel radiation therapy schedule based on a mathematical model of cell-state plasticity. Randles et al. (59) applied a computational model of the spatiotemporal dynamics of the perivascular niche to optimize the standard-of-care treatment for GBM. Brüningk et al. (60) proposed a personalized treatment strategy using intermittent radiotherapy based on a mathematical model of tumor growth, radiation response, and a patient-specific evolution of resistance. Our modeling approach is more modest because our data are limited to surveillance MRI.

### 4.2 Rationale for our scientific approach and potential refinements

The ASU model has three advantages over contemporary machine-learning methods. First, unlike a neural network that may contain millions of parameters that must be fitted, the ASUB model has only seven (**Table 2**). Second, it is possible to quantify the uncertainties in the output of the ASUB model and prove that it always produces nonnegative solutions (47). (Put another way, the ASUB model cannot “hallucinate,” unlike some machine-learning models.) Finally, only modest computing resources are needed: a full set of 60-day simulations takes less than two minutes on a modern multicore CPU.

The patients in our data set received a variety of treatments, including radiation, chemotherapy, antiangiogenic drugs, and additional surgery. Except for surgery, which reduces tumor cell populations beyond the scope of the model, we have not attempted to account for treatment timelines. The model’s growth and quiescence terms, together with their parameterizations, reflect the sum total of treatment response and resistance. As explained in the introduction, we simply do not have a sufficiently validated understanding of GBM tumor biology or associated patient data to include more detailed mathematical descriptions of the effects of specific treatments. Instead, we lump treatment effects and tumor biology into one set of net diffusion, growth, and quiescence terms. We take the view that the treatment response of recurrent GBM tumors is unpredictable, and, analogously to simulations of portfolio performance for financial planning, have identified ranges of model parameters that yield clinically representative scenarios for patient counseling.

Nevertheless, a future (and larger) study might stratify patients according to selected biomarkers. For example, MGMT (O^6^-methylguanine (O^6^-MeG)-DNA methyltransferase) promoter methylation status (61) and isocitrate dehydrogenase (IDH) mutations (62) can affect treatment outcomes in GBM. Modified ranges of model parameters may yield more clinically relevant results for selected subsets of patients.

### 4.3 Potential integration with clinical workflows

Once the MR scans from a given patient have been uploaded to the appropriate digital repository, the preprocessing workflow outlined in Section 3.1 can be applied. The ASUB model is not sensitive to small changes in segmentation boundaries; artificial intelligence or other semi-automated method could be used to generate a preliminary segmentation, as long as there are no gross errors in identifying the tumorous regions. The resulting preprocessed data can be downloaded as any other imaging files, and the simulation program itself could be packaged as an “app” on a physician’s laptop. The simulations used to generate outputs like **Figure 2** can be run in a couple of minutes.

### 4.4 Sources of errors and failure analysis

Co-registration errors are unavoidable and, in some cases, significant. One example is in **Figure 5**. The tumor is simulated using the computational domain in panel (f). However, in panel (g), the cyan curve representing the boundary of the edematous region for the “best” simulated tumor runs through the resection cavity in the subsequent scan. Panel (h) shows the corresponding SPM12-derived brain segmentations. Differences from panel (f) in the size and placement of the ventricles and gray- and white-matter tracts are evident. Measures of tumor agreement and containment must be interpreted in light of such geometric differences in the reconstructed domains.

The effect of co-registration errors can be especially important in the vicinity of the resection cavity, and tumor-related changes to the brain geometry may be a contributing factor. Our model simulations do not account for mass effect, and a future effort may benefit from including one (but would involve significant additional computational complexity).

Although T1 and T2 sequences were used for each patient, no other uniform imaging protocol has been followed. The data used in this research project were collected on different scanners. The strength of the magnetic field varies, and there are differences in the way that operators positioned the patients. Such variations complicate image comparisons, but they also reflect “real world” clinical data. We believe that our approach will be practicable in contemporary routine clinical settings where uniform, prospective scanning protocols may not exist.

The ASUB model and the FKPP model presume a fixed carrying capacity for tumor cells. When initialized with cell densities below the carrying capacity, neither model can simulate a decreasing cell population. When proliferating cells become sufficiently numerous in the ASUB model, they join the quiescent pool. Sufficiently dense regions of quiescent cells are assumed not to enhance on T1 imaging (they are assumed to form a hypointense region). Thus, our existing model parameterizations cannot always simulate a rapid reduction in the number of enhancing voxels. There are 4 cases in which $R^{\text{best}}$ *>*100%, of which **Figure 5** is one, and each involves a significant (*>* 66%) reduction in the number of enhancing voxels between the initial and subsequent scans. This result may simply reflect an inherent limitation of the ASUB model. Future work (and a larger dataset) will be needed to get a better idea of how often simulation failures like that in **Figure 5** are likely to occur and to understand the issues in model initialization and parameterization that lead to such failures.

Our methods for initializing tumor cell populations and performing comparisons with patient scans are simplistic, and the tumor segmentations are based solely on relative pixel intensity. Some patients in our data set were treated with anti-angiogenic agents, which may affect the appearance of tumor on MRI (63) (“pseudoresponse”). Radiotherapy can cause “pseudoprogression,” where dying cells cause imaging enhancement that can be difficult to distinguish from actively growing tumor cells (64, 65). The addition of perfusion MRI may improve our characterization of treatment effects and provide better initializations of the model.

Finally, it may be possible to improve the agreement and containment measures with a more sophisticated model of cell motility than isotropic diffusion. Diffusion tensor imaging (DTI)—either of individual patients or by applying an averaged DTI map over many patients—may be useful in reaction-diffusion models of glioma progression (66). Analogous approaches involve viscous stress tensors (67). In addition, the traveling-wave characteristics of the Fisher and ASU-Barrow models may allow better initialization of tumor cell populations in edematous regions (48, 52).

## Data Availability

The datasets for this article are not publicly available due to concerns regarding participant/patient anonymity. Requests to access the datasets should be directed to the corresponding authors.
